# N‐Lactoyl Amino Acids: Emerging Biomarkers in Metabolism and Disease

**DOI:** 10.1002/dmrr.70060

**Published:** 2025-06-18

**Authors:** Khaled Naja, Laila Hedaya, Asma A. Elashi, Manfredi Rizzo, Mohamed A. Elrayess

**Affiliations:** ^1^ Biomedical Research Center QU Health Qatar University Doha Qatar; ^2^ School of Medicine Promise Department University of Palermo Palermo Italy; ^3^ Department of Medicine Ras Al Khaimah (RAK) Medical and Health Sciences University Ras Al Khaimah UAE; ^4^ College of Medicine QU Health Qatar University Doha Qatar

**Keywords:** Lac‐Phe, lactate, metabolite, N‐lactoyl amino acids

## Abstract

N‐lactoyl amino acids (Lac‐AA) form an emerging class of metabolites that have gained significant attention in recent years due to their ubiquitous presence in different biological systems and potential roles in various biochemical processes. This narrative review aims to provide a comprehensive overview of the current understanding of Lac‐AA, emphasising their biosynthesis, physiological roles, and potential implications in various diseases. We discuss the discovery of Lac‐AA as signalling molecules, and their involvement in exercise‐induced appetite suppression, energy metabolism, and other pathways. This review explores the complex relationship between Lac‐AA and various pathological conditions, including mitochondrial disorders, type 2 diabetes, phenylketonuria, cancer, and rosacea. We also examine the interplay between Lac‐AA and the gut microbiota, as well as their association with metformin treatment. Furthermore, we address the ongoing debate regarding whether Lac‐AA are merely reflections of lactate and amino acid levels or independent signalling molecules. This review synthesises the latest research findings, highlights the significance of Lac‐AA in metabolic research, and identifies promising avenues for future investigation in this rapidly evolving field.

## 1 Introduction

The human metabolome is a vast and complex landscape, encompassing a multitude of metabolic pathways and interactions that are not yet fully understood. These interactions and pathways are constantly adapting and evolving in response to both internal and external stimuli. N‐lactoyl‐amino acids (Lac‐AA), a class of metabolites formed by enzymatic conjugation of lactate to an amino acid via a peptide bond, have garnered significant attention in recent years due to their pervasive presence in mammalian systems and their potential implications for various biological processes [1]. Their increasing recognition in recent research underscores their potential roles in metabolism and disease mechanisms. Lac‐AA demonstrate a strong positive correlation with one another [2, 3], suggesting that they act as a cohesive family. N‐lactoyl‐phenylalanine (Lac‐Phe), a conjugate of lactate and phenylalanine, is the most abundant and representative member of the Lac‐AA family.

In earlier metabolomic studies, Lac‐AA have been reported as unknown compounds with X‐identification numbers (ID) or misannotated as 1‐carboxyethyl modified amino acids [2]. Therefore, they are often overlooked or incorrectly interpreted in the context of metabolic pathways and disease associations. Recent advancements in metabolomic techniques and improved annotation methods have shed light on the true identity of these compounds. Leading metabolomics companies have now confirmed that the metabolites previously identified as 1‐carboxyethyl modified amino acids are, in fact, N‐lactoyl modified amino acids (Table 1). Accordingly, in this review, we will consistently refer to and treat these metabolites as N‐lactoyl amino acids rather than 1‐carboxyethyl modified amino acids. This approach will ensure clarity and accuracy in discussing the biological significance, metabolic pathways, and potential clinical implications of Lac‐AA in various physiological and pathological contexts. Relevant literature was identified through comprehensive searches of PubMed, Scopus, and Google Scholar, and all pertinent studies published up to the present were included based on their significance to the review's objectives and thematic relevance.

**TABLE 1:** Correction of N‐lactoyl‐amino acids mislabelling.

Unknown ID	Incorrect identification	Correct identification	Human metabolome database ID
X‐15497	1‐carboxyethylphenylalanine	N‐lactoyl phenylalanine	HMDB0062175
X‐13529	1‐carboxyethylvaline	N‐lactoyl valine	HMDB0062181
X‐18889	1‐carboxyethylleucine	N‐lactoyl leucine	HMDB0062176
X‐22102	1‐carboxyethylisoleucine	N‐lactoyl isoleucine	HMDB0062180
X‐19561	1‐carboxyethytryrosine	N‐lactoyl tyrosine	HMDB0062177
X‐25607	1‐carboxyethylhistidine	N‐lactoyl histidine	—
—	—	N‐lactoyl tryptophan	HMDB0062178

The discovery of Lac‐AA highlights the ongoing exploration of the human metabolome and the potential for identifying novel compounds with biological significance. This review aims to provide a comprehensive overview of the current understanding of N‐lactoyl amino acids, focusing on their biochemical pathways, physiological roles, and implications in various pathological conditions. By synthesising the latest research findings, we seek to highlight the significance of these compounds and identify promising avenues for future investigation.

## 2 Brief History

Lac‐AA were first discovered in 2009 in the food industry as being formed during fermentation of Parmigiano‐Reggiano cheese, which is renowned for its complex flavour profile and texture [4]. Additional research in this field has characterised Lac‐AA as a taste‐enhancer substances which are produced from free amino acids and lactic acid through the enzymatic activity of lactoyl transferase in *Lactobacillus* species, especially in soy sauce and meat products [5, 6, 7, 8]. Recently, Lac‐AA have emerged in the field of biotechnology, particularly in the field of cell culture and biotherapeutic production, demonstrating their potential as a promising alternative to traditional amino acids. By demonstrating an improved solubility compared to their parent amino acids, these innovative compounds may lead to the development of more effective and concentrated cell culture media formulations, thereby enhancing the production of next‐generation biotherapeutics, which could be advantageous in certain applications [9]. The first identification of Lac‐AA in mammals was reported by Jansen et al. in 2015 [1], marking a significant advancement in the field. Since then, research has expanded on these metabolites, trying to uncover their widespread presence and significant physiological relevance.

## 3 Biosynthesis, Transport and Excretion

The pioneering study of Jansen et al. [1] unveiled Lac‐AA as a previously unrecognised class of metabolites synthesised through the reverse proteolysis of lactate and amino acids and catalysed by the enzyme cytosolic nonspecific dipeptidase 2 (CNDP2), challenging the long‐held belief that reverse proteolysis is negligible in vivo. Furthermore, they showed that the formation of Lac‐Phe necessitates the presence of cells, as incubating lactate and phenylalanine with plasma alone did not result in Lac‐Phe production. Thereafter, Li et al. [10] confirmed that CNDP2 is a principal biosynthetic enzyme responsible for basal and exercise‐inducible Lac‐Phe production in vivo. Although these findings implicate a mammalian origin, CNDP2‐deficient mice display only a partial reduction in Lac‐Phe [11], suggesting that other pathways may also contribute to its biosynthesis, possibly including microbial production.

Indeed, in this context, Bottesini et al. [12] demonstrated that Lac‐Phe can be produced in vitro by the yeast peptidase carboxypeptidase Y. Moreover, Sgarbi et al. [13] reported the ability of viable *Lactobacillus helveticus* and *Lactobacillus rhamnosus to form Lac‐Phe.* Interestingly, both strains were capable to synthesise the metabolite even without lactate addition but only adding phenylalanine. More recently, Yang et al. [14] showed that the swine‐derived *Ligilactobacillus salivarius* is also able to synthesise this metabolite. Lac‐Phe is also a phytochemical component of the *Phyllanthus emblica* plant, indicating that this metabolite can be produced across a diverse range of biological systems [15]. It is important to note that while gut microbiota can potentially produce Lac‐AA, the in vivo contribution of this pathway to overall Lac‐AA levels has not been definitively established. Further research is needed to quantify the relative contributions of host and microbial sources to Lac‐AA production in vivo.

Basal levels of Lac‐AA, particularly Lac‐Phe, can be detected in the circulation even under resting physiological conditions, which appear to maintain an equilibrium between Lac‐Phe and the pool of lactate and phenylalanine [1, 2, 10, 11]. Thus, Lac‐AA production might be further enhanced by factors that influence the metabolism of their precursors, especially lactate, which is a common backbone for all members of the family. For instance, when the cellular demand for ATP exceeds the available supply, such as in the case of hypoxia, lactate production is stimulated due to the activation of the glycolysis pathway and the reduction of pyruvate into lactate instead of entering the mitochondria for oxidative phosphorylation [16]. Alternatively, cellular reprograming may introduce a metabolic shift into aerobic glycolysis leading to elevated lactate levels even in normoxia, a phenomenon that is widely observed in rapidly proliferating cells such as activated immune cells, pluripotent embryonic stem cells and cancer cells [17]. While lactate plays important physiological roles by itself, it is removed from the circulation upon need and can be metabolised to pyruvate or used up for gluconeogenesis. It can also act as a donor in lysine lactylation, a posttranslational modification, further extending its functional range [18]. Similarly, the accumulation of lactate in some cases such as strenuous exercise, infection, or cancer may enhance the synthesis of Lac‐AA, serving new biological roles in both health and disease [10, 19].

Although in vitro studies showed extreme resistance of Lac‐Phe in laboratory settings mimicking the human gastrointestinal tract [20], this pseudopeptide metabolite has been found to be completely ineffective when dosed orally [10]. The discrepancy between in vitro stability and in vivo efficacy highlights the importance of considering factors such as absorption, distribution, and metabolism when evaluating the potential therapeutic applications of Lac‐AA.

Using vesicular transport assays, Jansen et al. [1] confirmed that ATP binding cassette subfamily C member 5 (ABCC5) transports Lac‐Phe, and therefore most likely also transports the other Lac‐AA. Very recently, Li et al. [21] demonstrated that the solute carrier family 17 member 1 (SLC17A1) and solute carrier family 17 member 3 (SLC17A3) are significant mediators of Lac‐Phe efflux in both mice and human kidney cells. Overexpression of these transporters in cultured cells resulted in increased Lac‐Phe levels in media, indicating their role as plasma membrane transporters for this metabolite. Additionally, by analysing genetic data from human populations, they found strong associations between urinary Lac‐Phe levels and polymorphisms within the *SLC17A1‐4* locus. Moreover, the genetic ablation of either SLC17A1 or SLC17A3 in mice led to a notable reduction in urinary Lac‐Phe levels, suggesting that these transporters are essential for the renal excretion process. However, plasma Lac‐Phe levels remained unchanged upon knockout of these transporters, indicating that it might be biochemically decoupled from the urinary Lac‐Phe pool. This also implies the existence of additional transporters responsible for Lac‐Phe efflux into other biological fluids and opens promising avenues for future research to identify and characterise these transporters.

## 4 N‐Lactoyl‐Amino Acids as Signalling Molecules

Lac‐AA have emerged as important blood‐borne signalling molecules with diverse physiological roles, particularly in the context of exercise, appetite, and energy metabolism. Mechanistically, Lac‐AA are thought to act as signalling metabolites via G protein‐coupled receptors (GPCRs). Particularly, Lac‐Phe has been demonstrated to activate specific GPCRs (GPR139, GPR147, GPR154, GPR100, GPR202) at physiologically relevant concentrations, indicating a potential mechanism for its signalling effects [22]. This activation occurs within a micromolar range, supporting the hypothesis of a local or paracrine signalling action. Interestingly, these receptors are implicated in a spectrum of neurophysiological functions. As a case in point, GPR139 is expressed in several areas of the central nervous system and is a putative inducer of calcium signalling [23]. It is thought to be involved in the regulation of different physiological and behavioural phenotypes, such as locomotor activity, body mass, glucose homoeostasis, substance abuse and sleep phases, in addition to its neuroprotective roles in neurodegenerative diseases [23, 24]. Another example is GPR154, currently named neuropeptide S receptor 1 (NPSR1), which appears to regulate behaviours related to substance abuse and cognitive functions and was associated with the pathophysiology of multiple diseases [25]. Generally, due to their involvement in a wide range of processes, research focused on GPCRs and Lac‐AA may unlock several insights into the mechanisms of Lac‐AA in health and disease.

In 2022, Li et al. demonstrated, in a leading‐edge study [10], that Lac‐Phe is an exercise‐induced metabolite that suppresses appetite and obesity by reducing food intake in mice, with similar effects observed in humans and racehorses. Specifically, they showed that a single intraperitoneal injection of Lac‐Phe can significantly reduce food intake by approximately 50% in diet‐induced obese mice. This hypophagic effect occurred without any indications of malaise and was not seen when similar doses of L‐lactate and phenylalanine were given individually. Additionally, the appetite‐suppressing effect of Lac‐Phe was not seen in lean mice. One possible reason could be the increased permeability of the blood‐brain barrier in obesity, easing the access of Lac‐Phe to the brain and signal transduction. Alternatively, altered signalling pathways and disruption of homoeostasis could affect the sensitivity of the brain to Lac‐Phe, leading to differential effects in obese mice. Notably, feeding mice a high fat diet for 28 days significantly elevates *Gpr139* mRNA expression in the hypothalamus, an effect which was not observed with shorter periods or standard chow [24]. In addition, knockdown of *Gpr139* in the arcuate nucleus of the hypothalamus, one of the regions activated by Lac‐Phe [26], leads to body mass gain [24]. Therefore, a plausible explanation for the lack of effect in lean mice is the insufficient GPR139 levels, which could be required to facilitate Lac‐Phe signalling for appetite suppression. Although food intake was not significantly affected by GPR139 depletion in this study, this experiment was performed on mice fed standard chow [24]. Considering the differential effect of diet type on GPR139, diet‐induced obese mice could constitute a better model for studying the effect of GPR139 on food intake and the role of Lac‐Phe in this process.

Further studies [27, 28] highlighted the role of Lac‐Phe as a key molecular mediator of the metabolic benefits of physical activity. Our recent study by Weber et al. [29] demonstrates that circulating Lac‐Phe levels are strongly and acutely influenced by exercise intensity. However, many studies have linked Lac‐Phe to a decreased physical performance. Very recently, Sellami et al. [30] reported that poor responders to exercise have higher levels of Lac‐AA, especially Lac‐Phe, compared to good responders. Additionally, the study observed a negative correlation between the expression of *CNDP2* and the percentage of slow‐twitch muscle fibres, highlighting the role of both Lac‐AA and CNDP2 in exercise physiology. In 2020, Nierenberg et al. [31] conducted a community‐based study on samples from middle‐aged adults, and identified Lac‐Phe as one of seven metabolites associated with declines in gait speed over the follow‐up period. Moreover, in 2023, Stein et al. [32] conducted a study examining the metabolomic profiles of soldiers in the United States army before entering the Special Forces Assessment and Selection course. This study aimed to identify differences in metabolic signatures between those who were ultimately selected and those who were not selected for Special Forces training. Lac‐Phe was one of the metabolites found to be significantly higher in non‐selected candidates compared to selected candidates. Furthermore, higher levels of Lac‐Phe correlated with both lower physical performance and worse diet quality. The association of Lac‐Phe with lower physical performance can be understood through the lens of metabolic priorities, where the benefits to metabolic health may come at the expense of physical performance.

The relationship between Lac‐Phe and metformin has emerged as a significant area of research, particularly in understanding the mechanisms underlying metformin's effects on weight loss and appetite suppression. Very recently, two pioneering studies [2, 11] have established the role of Lac‐Phe as a key downstream mediator of the effects of metformin on energy balance. Metformin inhibits mitochondrial complex I, leading to a shift towards glycolysis and increased intracellular lactate production. This increased lactate flux drives the biosynthesis of Lac‐Phe through mass action. Xiao et al. [11] demonstrated that metformin is a strong pharmacological inducer of Lac‐Phe levels in both mice and humans in a CNDP2‐dependent manner in macrophages and gut epithelial cells. Moreover, they showed that CNDP2 knockout mice, which are resistant to metformin‐inducible increase in Lac‐ Phe, are also resistant to the drug's anti‐obesity effects. Relatedly, an in silico study [33] revealed that two anti‐obesity strategies, treatment with *trans*‐10 and *cis*‐12 conjugated linoleic acid and caloric restriction, increase *Cndp2* expression in adipose tissue in animal models. This warrants further investigation into whether CNDP2‐mediated formation of Lac‐AA and the subsequent decrease in food intake underlies the anti‐obesity effects of these strategies.

Further validating the potential function of Lac‐AA in appetite regulation, Scott et al. [2] indicated that food intake has a considerable influence on Lac‐AA levels. For example, they observed that Lac‐Phe serum levels in fasting type 2 diabetes patients were significantly higher than in those who had eaten less than 6 h prior to sample collection. Another interventional study revealed a significant increase in Lac‐Phe levels post‐prandially [2], suggesting that Lac‐Phe elevation may represent part of a feedback mechanism between food intake and appetite. Consistent with this, acute Lac‐Phe treatment in mice activates several neuronal populations in the hypothalamus and brainstem [26], two regions that have well‐established roles in appetite regulation and energy balance [34]. However, further studies are required to characterise the specific neuronal types activated, as well as the downstream molecular and physiological effects of their activation by Lac‐Phe and possibly other Lac‐AA.

Lac‐Phe levels can be stimulated with diverse types of diets [2], though likely through different mechanisms. For example, while high‐sugar mediated induction of Lac‐Phe may reflect a glycolytic status where lactate concentration is increased, habitual or mixed meals that are high in protein content may additionally increase the concentration of amino acids that can be conjugated with lactate to form Lac‐AA. As high‐protein diets increase satiety, leading to decreased body weight and fat mass loss, more studies are required to determine whether Lac‐AA are involved in this process [35]. In contrast, mice subjected to ketosis stimuli, such as a ketogenic diet or a ketone ester drink, did not experience any significant change in Lac‐Phe plasma levels upon these interventions, probably due to the lack of stimulation of lactate and phenylalanine [26]. Interestingly, Mathew et al. [36] showed that the post‐meal increase in Lac‐Phe was significantly different between solid and liquid meal intake. Their study reported a 220% increase in Lac‐Phe in response to both date fruit varieties, while the response to the liquid glucose challenge, though significant, resulted in a much lower increase (37%). This varied response indicates that Lac‐Phe may play a distinct regulatory role in appetite depending on whether the meal is solid or liquid. This could provide valuable insights into how different food forms influence hunger signalling.

Lac‐AA were also found to be involved in other signalling pathways and demonstrated potential for broader applications in metabolic research and therapeutic development. Very recently, Zhang et al. [37] reported an association between sleep patterns and Lac‐AA, as exposures and outcomes, respectively. Specifically, these metabolites were associated with sleep duration, sleep disordered breathing, sleep timing, and heart rate during sleep. While not directly studied, altered Lac‐AA levels could be explained by sleep phenotype‐induced changes in the abundance of their precursors. For instance, cortical lactate concentration in male mice was found to rise and stay high during natural and enforced wakefulness as well as rapid eye movement sleep, probably due to high activity states where the demand for lactate as an energy substrate increases [38]. Moreover, later sleep timing was associated with higher plasma levels of branched chain amino acids in human subjects [39]. Similarly, peripheral metabolic profiling of patients with insomnia revealed perturbations of the glycolytic pathway, including a decrease in lactate concentration, in addition to altered branched chain amino acid levels compared to good sleepers [40]. Some of these metabolites exhibited time‐dependent fluctuations across day and night, reflecting circadian‐regulated metabolic activity and underscoring the importance of considering temporal factors when studying Lac‐AA. Moreover, serum lactate concentration is increased in patients with obstructive sleep apnoea, which may reflect a glycolytic pathway activation due to intermittent hypoxia, a fundamental characteristic of the disorder [41]. Collectively, these studies suggest a potential role of Lac‐AA as a biomarker whose study can be valuable for understanding sleep‐related disorders and associated metabolic alterations. Some of the Lac‐Phe activated GPCRs, namely GPR139, GPR147 and GPR154, were previously shown to influence sleep and wakefulness [42, 43]. Thus, studying them along with Lac‐AA could reveal new mechanisms of sleep regulation.

The role of Lac‐AA as signalling molecules was also demonstrated by Liu et al. [44] who used Lac‐Phe as a key component in the bone layer of the biphasic scaffold. Loaded onto the silk fibroin methacryloyl coating of a 3D‐printed bioactive glass scaffold, Lac‐Phe played a crucial role in regulating the osteogenic microenvironment. It enhanced microvascular endothelial cell activities, promoted bone marrow mesenchymal stem cell recruitment, and directed their differentiation towards osteogenesis. By incorporating Lac‐Phe, the researchers created a more favourable environment for bone regeneration, contributing significantly to the overall enhanced osteochondral regeneration observed in their study. In another study by Kovaničová et al. [45], N‐lactoyl tryptophan was found to correlate with both the level of cold acclimatization and changes in thyroid and parathyroid hormones following cold exposure. This suggests its potential role in the metabolic adaptations associated with cold stress. Despite ongoing research, there remain numerous unanswered questions concerning the precise role of Lac‐AA as signalling molecules in various biological processes, and many future investigations are warranted.

## 5 N‐Lactoyl‐Amino Acids in Diseases: Potential Biomarkers or Protective Metabolites?

The implications of Lac‐AA as signalling molecules extend beyond basic physiology, indicating their potential roles in various pathological conditions. Recent research highlights the significance of Lac‐AA as potential biomarkers and therapeutic targets in various diseases (Table 2). These metabolites are increasingly recognized for their involvement in metabolic processes and disease mechanisms. Recognising Lac‐AA as biomarkers is crucial for early disease detection and personalised treatment because their levels correlate strongly with the onset and severity of various conditions. While the associations between Lac‐AA and various pathological conditions are intriguing, it is paramount to recognise that most existing research has established correlational rather than causal relationships. Further research is essential to elucidate whether these metabolites actively influence disease processes or merely serve as biomarkers reflecting the current state of conditions such as hypoxia.

**TABLE 2:** Association between N‐lactoyl‐amino acids and various conditions and diseases.

Metabolite	Associated disease/condition	Regulation (↑/↓)	Biological sample type	References
Lac‐Phe	Type 2 diabetes	↑	Serum and plasma	[2, 46, 47, 48]
Lac‐Leu				
Lac‐Ile				
Lac‐Tyr				
Lac‐Val				
Lac‐Phe	Insulin resistance	↑	Serum	[48, 49, 50]
Lac‐Phe	Diabetic retinopathy	↑	Plasma	[51]
Lac‐Ile				
Lac‐Tyr				
Lac‐Val				
Lac‐Leu	History of gestational diabetes	↑	Serum	[52]
Lac‐Ile				
Lac‐Val				
Lac‐Ile	Retinopathy of prematurity	↑	Serum	[53]
Lac‐Ile	Metabolic dysfunction‐associated steatotic liver disease	↑	Plasma	[54]
Lac‐Val				
Lac‐Phe	Mitochondrial dysfunction	↑	Plasma	[3, 19, 55]
Lac‐Leu				
Lac‐Ile				
Lac‐Tyr				
Lac‐Val				
Lac‐Phe	Phenylketonuria	↑	Serum and plasma	[1, 56]
Lac‐Leu	Metabolic syndrome in people living with HIV	↑	Plasma	[57, 58]
Lac‐Ile				
Lac‐Phe	Rosacea	↑	Plasma	[59]
Lac‐Leu	Colorectal cancer	↓	Urine	[60]
Lac‐Val				
Lac‐Tyr	Incidence of cardiovascular diseases	↑	Plasma	[61]
Lac‐Leu	Left ventricular diastolic dysfunction	↑	Serum	[62]
Lac‐Ile				
Lac‐Val				
Lac‐Phe	COVID‐19 severity	↑	Plasma	[63]
Lac‐Phe	Primary open angle glaucoma	↓	Plasma	[64]
Lac‐Phe	Future risk of glioma	↑	Plasma	[65]
Lac‐Leu				
Lac‐Val				
Lac‐Trp	Osteoporosis	↑	Serum	[66]
Lac‐Phe	Quantitative interstitial abnormalities in smokers	↑	Plasma	[67]
Lac‐Tyr				

Abbreviations: COVID‐19, coronavirus disease 2019; HIV, human immunodeficiency virus; Lac‐Ile, N‐lactoyl isoleucine; Lac‐Leu, N‐lactoyl leucine; Lac‐Phe, N‐lactoyl phenylalanine; Lac‐Trp, N‐lactoyl tryptophan; Lac‐Tyr, N‐lactoyl tyrosine; Lac‐Val, N‐lactoyl valine.

**FIGURE 1:**
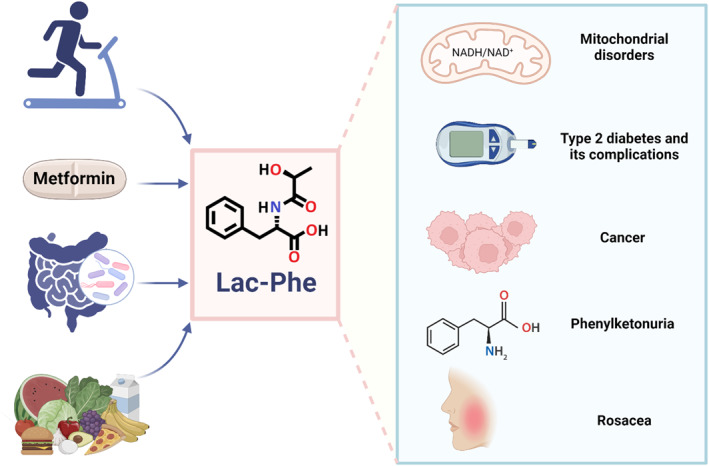
Potent inducers of N‐lactoyl amino acids, and main diseases associated with an increase in its levels. N‐lactoyl phenylalanine (Lac‐Phe) production increases after exercise, metformin treatment, feeding, and by gut microbiota. Lac‐Phe is associated with various health conditions; it serves as potential biomarkers for mitochondrial disorders and septic shock, shows a complex relationship with type 2 diabetes and its complications, is significantly elevated in patients with phenylketonuria, exhibits altered levels in different types of cancer, and has a causal association with rosacea. Created in BioRender.

Figure 1 summarise the main diseases associated with Lac‐AA as well as potential sources of these metabolites.

### 5.1 Mitochondrial Disorders

In 2021, Sharma et al. [3] investigated the biochemical underpinnings of mitochondrial disorders, particularly focusing on the m.3243 A > G mutation, which is associated with diseases like mitochondrial encephalomyopathy lactic acidosis and stroke‐like episodes (MELAS). The study employed advanced proteomic and metabolomic techniques to identify biomarkers that correlate with disease severity. The levels of various Lac‐AA were significantly elevated in patients with this mutation compared with healthy controls, and correlated strongly with other markers of disease severity, suggesting a potential role as biomarkers of nicotinamide adenine dinucleotide (NADH) reductive stress associated with mitochondrial dysfunction. Afterwards, Xia et al. [55] demonstrated that Lac‐Phe has a strong coregulation with a myriad of metabolic indicators of afflicted mitochondrial function. Recently, Rogers et al. [19] reported higher levels of Lac‐Phe in septic shock patients. Interestingly, Lac‐Phe was even *more effective* than lactate in distinguishing between survivors and non‐survivors within 24 h of intensive care unit admission. The study emphasised the role of Lac‐Phe as a strong indicator of mitochondrial dysfunction, which may serve as a valuable biomarker for assessing the severity of septic shock. Nonetheless, as research progresses, Lac‐AA could deepen our understanding of mitochondrial pathologies and inform more effective treatment strategies.

### 5.2 Type 2 Diabetes, Diabetic Complications and Insulin Resistance

Recent studies have revealed a complex relationship between Lac‐AA and type 2 diabetes (T2D). While multiple studies reported significantly higher levels of Lac‐AA in T2D compared to prediabetes and non‐T2D controls [2, 47], Scott et al. [2] suggested that the difference is primarily driven by metformin treatment rather than T2D status alone. Relatedly, a pharmacometabolomic study indicated that Lac‐Phe is linked to a poor response to metformin in patients with T2D [68], which may reflect higher doses of metformin administered to non‐responders. However, this could also indicate worsened metformin response in patients with elevated Lac‐AA levels. Additional studies showed that, N‐lactoyl valine and N‐lactoyl isoleucine were positively correlated with haemoglobin A1C (HbA1C) in individuals subjected to bariatric surgery [69]. Moreover, Lac‐AA were found to be lower in breastfeeding mothers and higher in those with a history of gestational diabetes mellitus, or diabetes during pregnancy [52]. Very recently, Sharma et al. [48] demonstrated that the progression from prediabetes to diabetes is mediated by Lac‐AA among other metabolites. Indeed, insulin resistance is a critical factor in the development of T2D, serving as a precursor that can manifest years before the onset of the disease [70]. Interestingly, Lac‐AA are strongly associated with insulin resistance [24, 49, 50, 71]. In fact, Diboun et al. [50] demonstrated that Lac‐Phe is among the top metabolites differentiating insulin resistance from insulin sensitivity in apparently healthy non‐obese individuals. Moreover, this metabolite remained higher in individuals classified as insulin resistant regardless of their physical activity levels [49].

Lac‐AA are also depicted in the image in the context of diabetes‐related complications. Fernandes et al. identified Lac‐AA as significant factors that increase the risk for diabetic retinopathy in patients with T2D by an estimated range of 28%–35% [51]. While there is no research specifically discussing a link between Lac‐AA and diabetic nephropathy, several single nucleotide polymorphisms (SNPs) in the *CNDP2* gene were associated with increased risk [72, 73]. For example, carriers of the CC genotype at the rs7577 SNP had lower *CNDP2* expression levels and higher risk of diabetic nephropathy [72]. Relatedly, a study which included 190 kidney transplants [74], classified Lac‐Phe as one of the top 6 metabolites strongly associated with an increased risk of graft failure. Furthermore, using a multi‐omics‐based approach, Yousri et al. [75] found two CpG sites that are significantly associated both with T2D and Lac‐AA. Interestingly, one CpG site maps to the three prime untranslated region of thioredoxin interacting protein (*TXNIP*), which has a known role in promoting the development of T2D [76]. This CpG site was also significantly associated with several SNPs, most of which are within the solute carrier family 2 member 1 (*SLC2A1*) gene locus which encodes a glucose transporter (GLUT1) that was previously found to be suppressed by TXNIP [77] but stimulated by metformin [78]. The other CpG site belongs to the 6‐Phosphofructo‐2‐Kinase/Fructose‐2,6‐Biphosphatase 2 (*PFKFB2*) gene, which was previously linked to glucose homoeostasis‐related traits and diabetic nephropathy [79]. Notwithstanding the aforementioned findings, the relationship between Lac‐AA and diabetes complications remains somewhat ambiguous and equivocal. While preliminary studies suggest potential links, the mechanisms by which these compounds may influence diabetes‐related complications are not yet fully understood and require further investigations.

On the other hand, other studies hold a conclusion against the causality of Lac‐AA in T2D and suggest a protective role of Lac‐AA. In their study, Li et al. [10] showed that chronic Lac‐Phe treatment not only reduces adiposity and obesity but also improves glucose homoeostasis, suggesting a causative role for this metabolite in improving metabolic health. Supporting this claim and providing a suggested mechanism, suppressing hypothalamic expression of GPR139, of which Lac‐Phe is a potential agonist, was shown to improve fasting blood glucose and insulin levels in mice [24]. In the same direction, a mediation study [80] showed that N‐lactoyl leucine may mediate the association between blood group A and insulin sensitivity and clearance. Additionally, although Lac‐Phe levels were reported to be significantly higher in acute myocardial infarction (AMI) patients with diabetes than in AMI patients without diabetes, differential correlation analysis revealed that HbA1C becomes inversely correlated with Lac‐Phe only in diabetes‐AMI. This raises the possibility of Lac‐Phe acting as a coping mechanism, rather than a causative factor of diabetes [55]. Considering the metabolic signature indicative of mitochondrial dysfunction in diabetes‐AMI, and the connection of Lac‐Phe with mitochondrial disease, the increase of Lac‐Phe could also be a response to oxidative stress and mitochondrial overload [3, 55].

Several studies highlighted the strong association of lactate with T2D and complications [81, 82, 83] and insulin resistance [82]. This association is also applicable to branched‐chain amino acids [84, 85, 86] and aromatic amino acids [85]. Although highly speculative, Lac‐AA may accordingly act as a ‘metabolic sink’ for these metabolites, potentially buffering the negative effects of lactate, BCAA, and aromatic amino acids on glucose homoeostasis.

Although the association between Lac‐AA and T2D is becoming clearer, further research is needed to fully understand their role in T2D pathophysiology and their potential as therapeutic targets or diagnostic markers. Ongoing studies are exploring the mechanistic pathways through which Lac‐AA exert their effects, as well as their interactions with common diabetes medications like metformin. Understanding these relationships could lead to more effective treatment strategies and improve clinical outcomes for individuals with T2D.

### 5.3 Phenylketonuria

In their pioneering study, Jansen et al. [1] also reported that Lac‐Phe levels were significantly elevated in patients with phenylketonuria (PKU) compared with healthy controls. This elevation correlates with increased plasma phenylalanine levels. Intriguingly, lactate levels remain comparable between PKU patients and healthy individuals. This could be attributed to the non‐altered pathways responsible for lactate production and clearance in PKU, maintaining a balance that results in similar lactate concentrations across both groups. Recently, Van Wegberg et al. [87] reported significantly higher levels of Lac‐Phe in PKU patients compared to control. This elevation is notable alongside other biomarkers like phenylalanine and glutamyl‐phenylalanine. Remarkably, Lac‐Phe demonstrated, in the latter study, a strong association with various cognitive functions outperforming traditional measures like phenylalanine in predictive models. A tentative mechanism could be the overstimulation of GPR139, which was shown to be activated by Lac‐Phe [22]. Since L‐phenylalanine, a precursor of Lac‐Phe, is a well‐established agonist of GPR139 [23], understanding the interplay between L‐phenylalanine, Lac‐Phe and GPR139 may yield valuable insights into the mechanisms of the PKU.

### 5.4 Cancer

The significant production of lactate by cancer cells was first observed by Otto Warburg in 1923 [88]. He noted that cancer cells exhibit increased glycolysis and produce excessive lactate even in the presence of oxygen, a phenomenon now known as the Warburg Effect. This observation has been consistently documented in cancer research, highlighting the metabolic alterations that characterise malignant cells. The relationship between Lac‐AA and cancer is highly dependent on the type of cancer and the exact member of the Lac‐AA family. Pancreatic adenocarcinoma cancer cells PANC‐1 and CFPAC‐1 showed a significant difference in N‐lactoyl‐methionine compared with normal pancreatic duct epithelial cells, H6c7 [89]. Urinary levels of N‐lactoyl leucine and N‐lactoyl valine were significantly higher in colorectal cancer patients compared with the control group [60]. Moreover, N‐lactoyl isoleucine levels were significantly elevated in clear cell renal cell carcinoma and serum of renal cancer patients compared with healthy control cells [90]. Furthermore, the overexpression of solute carrier family 39 member 1 (SLC39A1) in renal cancer cells, specifically in the OSRC‐2 cell line, resulted in a significant downregulation of Lac‐Phe levels [91]. The role of CNDP2 in cancer is controversial. CNDP2 was shown to act as a functional tumour suppressor in gastric cancer [92]. Conversely, increased CNDP2 protein expression has been observed in colon cancer [93] and ovarian cancer [94], indicating that it may also contribute to tumourigenesis in certain contexts. Continued exploration into Lac‐AA is essential for advancing our understanding of their roles in cancer biology.

### 5.5 Rosacea

In a recent Mendelian randomisation study [59], Lac‐Phe was identified as having a statistically significant causal relationship with rosacea, suggesting that increased levels of this metabolite may contribute to the onset or worsening of the condition. While Lac‐Phe levels are elevated by various stimuli that increase lactate—such as exercise, metformin, and feeding—the rise of Lac‐Phe in rosacea, a skin disorder, is particularly intriguing. The exact mechanisms by which Lac‐Phe influences rosacea are not fully understood. However, the same study hypothesised that Lac‐Phe may affect neurovascular function, which is critical in the pathophysiology of rosacea. Neurovascular dysfunction can lead to increased vasodilation and inflammatory responses, typical features of rosacea [95]. However, previous studies [96] did not identify any significant role for phenylalanine in abnormal amino acid metabolism associated with the neurovascular reactivity of rosacea, while glutamic acid and aspartic acid were found to have important functions.

## 6 N‐Lactoyl‐Amino Acids and Gut Microbiota

Gut microbiota consists of trillions of microorganisms residing in the human gastrointestinal tract. These microbes play a crucial role in various metabolic processes, including the metabolism of lactate and amino acids. Recently, Yang et al. [14] featured the significance of Lac‐AA as metabolites produced by the swine‐derived *L. salivarius*, a bacterial species that is native to the human gut, highlighting their notable role in the probiotic functions of this bacterium. Moreover, He et al. [97] identified Lac‐Phe as a key metabolite that links the adverse effects of the Fusarium genus within the mycobiome to insulin sensitivity. Specifically, they observed higher levels of Lac‐Phe in individuals with impaired mitochondrial function, which are linked to disturbances in gut fungi and could have implications for metabolic diseases.

In 2019, Wilmanski et al. [98] presented significant findings regarding the relationship between blood metabolites and gut microbiome diversity, which has implications for human health monitoring and disease prediction. Interestingly, Lac‐Phe was classified among the strongest predictors of Shannon *α*‐diversity, indicating that individuals with higher levels of this metabolite tend to exhibit reduced microbial diversity. Lower diversity may correlate with various health issues, including metabolic disorders and inflammatory conditions. Noteworthy, the crosstalk between gut microbiota metabolism and metformin is now well established, and it is now clear that the gut microbiota participates in both the therapeutic and side effects of metformin [99]. Given the induction of Lac‐Phe by metformin, it is plausible that Lac‐Phe serves as a link between metformin treatment, the associated changes in gut microbiota, and the overall effects of metformin.

## 7 N‐Lactoyl‐Amino Acids: Just a Reflection of Lactate and Amino Acids?

**FIGURE 2:**
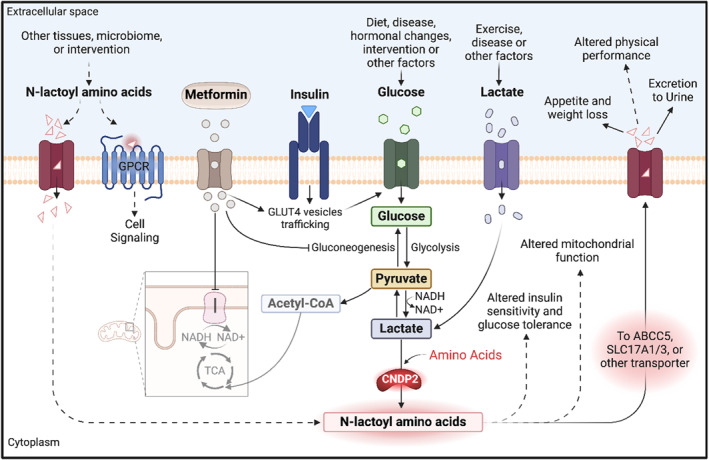
Possible mechanisms of N‐lactoyl amino acid regulation and their potential downstream effects. In mammalian cells, Lac‐AA are mainly synthesised through CNDP2‐catalysed interaction between lactate and the corresponding amino acids. The increase in intra‐ or extra‐cellular flux of lactate drives this process. When lactate is produced by skeletal muscles or other cells and accumulates in the bloodstream due to exercise or specific diseases, extracellular flux might be utilised. On the other hand, intracellular flux is employed upon treatment with metformin, which inhibits mitochondrial complex I in intestinal epithelial cells, increasing the NADH/NAD^+^ ratio and consequently, accumulating intracellular lactate. When synthesised, Lac‐AA may affect intracellular signalling pathways or be excreted from the cell via transporter proteins, such as ABCC5 and SLC17A1/3. While ABCC5 is ubiquitous and may transport Lac‐AA expressed in different tissues, SLC17A1/3 are primarily localised to the kidneys and mediates the renal excretion of Lac‐Phe into urine. When exported to the bloodstream, Lac‐Phe can be distributed to various organs, and act as a signalling molecule by regulating appetite and body weight. Moreover, chronic levels of Lac‐Phe have been shown to improve glucose tolerance in mice, though the effects on humans remain to be fully elucidated. Lac‐AA may also influence physical performance, although functional studies are needed to confirm a causal relationship, especially that lactate is a potential confounder. The association between Lac‐AA and mitochondrial dysfunction hints at a possible role of Lac‐AA in mitochondrial physiology, which warrants further research to substantiate a causal link. Recent evidence has shown that Lac‐Phe could activate GPCRs, opening new doors for studying Lac‐Phe as a signalling molecule and its downstream signalling pathways. ABCC5, ATP binding cassette subfamily C member 5; Acetyl‐CoA, acetyl coenzyme A; CNDP2, cytosolic nonspecific dipeptidase 2; GLUT4, glucose transporter type 4; GPCRs, G‐protein coupled receptors; Lac‐AA, N‐lactoyl amino acids; Lac‐Phe, N‐lactoyl phenylalanine; NADH/NAD^+^, reduced/oxidised nicotinamide adenine dinucleotide ratio; SLC17A1/3, solute carrier family 17 members 1 and 3; TCA, tricarboxylic acid. Created in BioRender.

The formation of Lac‐AA is thermodynamically favoured when there are high concentrations of lactate and amino acids present. The apparent equilibrium constants for Lac‐AA formation are higher than those reported for reverse proteolysis of regular amino acids [1], allowing Lac‐AA levels to approach micromolar concentrations in plasma when intracellular lactate and amino acid levels are high. Not surprisingly, an increase in the levels of Lac‐AA is observed in all scenarios where lactate and/or amino acids are high. Strikingly, Lac‐AA are predominantly formed from branched‐chain amino acids and aromatic amino acids, which are hydrophobic in nature. This is consistent across various studies and has important implications for our understanding of these metabolites. The observation that the water solubility of Lac‐AA is much higher than their corresponding amino acids [9], raises an intriguing question about why the formation of Lac‐AA appears to be selective for hydrophobic amino acids when the lactate conjugation increases solubility. A plausible mechanism is that the increased solubility may serve specific biological purposes, such as facilitating transport or signalling roles. Additionally, it could function as a regulatory mechanism for the concentrations of lactate or certain amino acids in specific cellular compartments or physiological states. A summary of possible mechanisms of Lac‐AA regulation and their potential downstream effects are illustrated in Figure 2.

## 8 Conclusion

As research on Lac‐AA continues to expand, it is becoming increasingly clear that these compounds play multifaceted roles in biological systems. Despite recent advances, the study of Lac‐AA is limited by methodological heterogeneity, a lack of standardized quantification protocols, and insufficient understanding of their precise physiological and pathological roles. It is important to note that the units and reporting methods vary across studies, with some reporting relative abundance and others providing absolute quantitation. This lack of standardisation can make direct comparisons between studies challenging. While mass spectrometry‐based methods offer the most universal approach for measuring Lac‐AA, there is still a need for standardized protocols and reporting to ensure consistency across studies and facilitate broader understanding of these metabolites in various biological contexts. Another point to consider is that the interconversion between lactate, amino acids, and Lac‐AA is very rapid. This fast interconversion can make it difficult to accurately measure intracellular levels, as effluxed Lac‐AA are continuously replenished from the large pool of lactate and amino acids. Additionally, the ineffectiveness of oral Lac‐Phe, attributed to its breakdown in the gastrointestinal tract, poses significant challenges for research by limiting its potential for oral administration in therapeutic applications. Moreover, Mechanistic uncertainties persist, with most disease associations remaining observational rather than causal. Future studies should focus on conducting mechanistic and longitudinal research to clarify their causal roles in health and disease, exploring their signalling pathways and interactions with gut microbiota, investigating their therapeutic potential and delivery strategies, and expanding research into their functions across diverse physiological, pathological, and environmental contexts such as exercise adaptation and metabolic disorders.

In conclusion, Lac‐AA are not merely metabolic intermediates, but they represent a fascinating aspect of biochemical processes that warrant deeper investigation. The ongoing debate regarding their importance and functionality highlights the complexity of the metabolic networks in which they are involved. This emphasises the critical need to advance our understanding in this area, as it is essential for unlocking their full potential in promoting human health.

## Author Contributions

K.N., L.H., A.A.E. and M.R. contributed to the writing of the manuscript. L.H. created the figures. M.A.E. revised the manuscript and supervised the work.

## Conflicts of Interest

The authors declare no conflicts of interest.

## Peer Review

The peer review history for this article is available at https://www.webofscience.com/api/gateway/wos/peer-review/10.1002/dmrr.70060.

## Data Availability

Data sharing not applicable to this article as no datasets were generated or analysed during the current study.
